# Diagnostic value of real-time polymerase chain reaction to detect viruses in young children admitted to the paediatric intensive care unit with lower respiratory tract infection

**DOI:** 10.1186/cc4895

**Published:** 2006-04-12

**Authors:** Alma C van de Pol, Tom FW Wolfs, Nicolaas JG Jansen, Anton M van Loon, John WA Rossen

**Affiliations:** 1 Department of Pediatrics, Division of Infectious Diseases, Wilhelmina Children's Hospital, University Medical Center Utrecht, Utrecht, The Netherlands; 2 Department of Pediatrics, Division of Intensive Care, Wilhelmina Children's Hospital, University Medical Center Utrecht, Utrecht, The Netherlands; 3 Department of Virology, Eijkman-Winkler Institute, University Medical Center Utrecht, Utrecht, The Netherlands

## Abstract

**Introduction:**

The aetiology of lower respiratory tract infections in young children admitted to the paediatric intensive care unit (PICU) is often difficult to establish. However, most infections are believed to be caused by respiratory viruses. A diagnostic study was performed to compare conventional viral tests with the recently developed real-time PCR technique.

**Method:**

Samples from children aged under 5 years presenting to a tertiary PICU suspected of having a lower respiratory tract infection were tested using conventional methods (viral culture and immunofluorescence) and real-time PCR during the winter season from December 2004 to May 2005. Conventional methods were used to check for respiratory syncytial virus, influenzavirus, parainfluenzavirus 1–3, rhinoviruses and adenoviruses. Real-time PCR was used to test for respiratory syncytial virus, influenzavirus, parainfluenzavirus 1–4, rhinoviruses, adenoviruses, human coronaviruses OC43, NL63 and 229E, human metapneumovirus, *Mycoplasma pneumoniae *and *Chlamydia pneumoniae*.

**Results:**

A total of 23 patients were included, of whom 11 (48%) were positive for a respiratory virus by conventional methods. Real-time PCR confirmed all of these positive results. In addition, real-time PCR identified 22 more viruses in 11 patients, yielding a total of 22 (96%) patients with a positive sample. More than one virus was detected in eight (35%) children.

**Conclusion:**

Real-time PCR for respiratory viruses was found to be a sensitive and reliable method in PICU patients with lower respiratory tract infection, increasing the diagnostic yield twofold compared to conventional methods.

## Introduction

Lower respiratory tract infections (LRTIs) cause significant hospitalization among children under 5 years old [1,2]. Although these infections are usually relatively mild, some children develop respiratory failure, necessitating mechanical ventilation and admission to the paediatric intensive care unit (PICU). The aetiology of (severe) LRTI is often difficult to establish. However, the majority of LRTIs in this young age group is believed to be caused by respiratory viruses. For the detection of respiratory viruses two conventional methods are commonly used: viral culture and direct immunofluorescence (DIF) assays. In addition, a third method based on molecular techniques has now become available, namely (real-time) PCR. Specifically, the real-time PCR format provides rapid results, within a clinically relevant period of time. It allows quantitative virus detection, and no post-PCR processing needs to be performed. Moreover, compared with standard format PCR, the risk for contamination is strongly reduced with real-time PCR, rendering false-positive results with real-time PCR highly unlikely [3].

Currently, there are no guidelines regarding which viral tests are appropriate for young children with LRTI in the PICU [4]. Conventional detection methods have several disadvantages compared with (real-time) PCR. Viral culture has been considered the gold standard for the detection of respiratory viruses, but its limitation is that it can only detect a small number. Furthermore, the yield of viral culture depends on the quality of sampling, the correct transport and storage of samples, and the type of cells used. In addition, the technique has a turnaround time of several days to weeks, and is therefore unable to guide initial patient management. The results of DIF assays are available more rapidly, and this has been shown to improve patient outcome with less antibiotic use and shorter hospital stay [5-7]. Unfortunately, the DIF assay is less sensitive than culture for the detection of certain pathogens.

PCR has been shown to be more sensitive than conventional techniques and is fast [8-11]. Additional advantages of PCR include its ability to identify multiple viruses simultaneously, and to detect viral and atypical pathogens that cannot be cultured or for which no DIF is commercially available (for example, coronaviruses, human metapneumovirus [hMPV], *Chlamydia pneumoniae *and *Mycoplasma pneumoniae*). Rapid and sensitive testing for a broad range of respiratory viruses is vital in the PICU, and it can improve our understanding of severe viral respiratory infections. Furthermore, it can be used to guide cohorting strategies that may protect other critically ill children and to guide initial therapy. Finally, evaluation of viral tests may be of future importance, when antiviral therapy becomes more widely available.

In the present study conventional methods (viral culture and DIF) were compared with real-time PCR for their ability to detect respiratory viruses in young children with LRTI admitted to the PICU. Secondary objectives were to describe the presence of viral pathogens and bacterial infection in LRTI patients in the PICU.

## Materials and methods

### Study population

Children aged under 5 years admitted to the PICU of the Wilhelmina Children's Hospital with LRTI were enrolled during one winter season from December 2004 to May 2005. Wilhelmina Children's Hospital is a tertiary university hospital with a 14 bed PICU facility, and serves as a referral centre for the central part of The Netherlands.

Patients were eligible if they had an admission diagnosis of (probable) bronchiolitis, (probable) pneumonia, or respiratory failure. Exclusion criteria were a primary cardiac or central origin of respiratory failure, and overt sepsis at the time of admission. The study described here was conducted as part of normal patient care; therefore, according to the Medical Ethical Research Council of our institution, there was no need for patient consent/ethical approval.

### Samples

Sputa and/or nasopharyngeal aspirates were obtained from all eligible patients for viral testing by viral culture, DIF and real-time PCR assays. In addition, sputum and blood samples (when available) were cultured and processed in accordance with standard microbiological procedures. Sputum samples were taken in a standardized manner, through sterile transtracheal aspiration in intubated children.

### Conventional virus detection

Some of the material obtained was used for immediate viral culture of RSV, influenzaviruses, parainfluenzaviruses (PIVs) 1–3, picornaviruses, adenoviruses and herpesviruses on LLC-MK2, RD, R-HELA and HEP-2C cells. Cultures were examined twice weekly for the development of a cytopathological effect. In positive cultures, virus was identified by immunofluorescence with monoclonal antibodies to RSV types A and B, influenzaviruses A and B, PIV 1–3 and adenoviruses (DaKo, Glostrup, Denmark). Rhinoviruses were identified using acid lability tests.

Some of the material was subjected to DIF assays to detect respiratory syncytial virus (RSV), influenzaviruses, PIV 1–3 and adenoviruses using Imagen kits (DaKo), in accordance with the manufacturer's recommended protocol. The remaining material was used for real-time PCR testing.

### Real-time PCR

Nucleic acids were extracted using the total nucleic acid protocol with the MagNA pure LC nucleic acid isolation system (Roche Diagnostics, Basel, Switzerland). Each sample was eluted in 200 μl buffer, which was sufficient for all real-time PCR analyses. cDNA was synthesized by using MultiScribe reverse transcriptase (RT) and random hexamers (both from Applied Biosystems, Foster City, CA, USA). Each 200 μl reaction mixture contained 80 μl of eluted RNA, 20 μl of 10 × RT buffer, 5.5 mmol/l MgCl_2_, 500 μmol/l of each deoxynucleoside triphosphate, 2.5 μmol/l random hexamer and 0.4 U of RNase inhibitor per microlitre (all from Applied Biosystems). After incubation for 10 minutes at 25°C, RT was carried out for 30 minutes at 48°C, followed by RT inactivation for 5 minutes at 95°C.

Detection of viral and atypical pathogens was performed in parallel, using real-time PCR assays specific for the following: RSV A and B, influenzavirus A and B, PIV 1–4, rhinoviruses, adenoviruses, human coronavirus OC43, NL63 and 229E, hMPV, *Mycoplasma pneumoniae *and *Chlamydia pneumoniae*. Real-time PCR procedures were performed as described previously [12-14].

Briefly, samples were assayed in duplicate in a 25 μl reaction mixture containing 10 μl (c)DNA, 12.5 μl 2 × TaqMan Universal PCR Master Mix (Applied Biosystems), 300–900 nmol/l of the forward and reverse primers and 75–200 nmol/l of each of the probes. All samples had been spiked before extraction with an internal control virus (murine encephalomyocarditis virus [RNA virus] and phocine herpes virus [DNA virus]) to monitor for efficient extraction and amplification, essentially as described previously [15].

### Clinical data collection and analysis

Each patient's medical records were reviewed for clinical data. Demographic data and the following clinical characteristics/outcomes were extracted and entered into standardized forms: gestational age, underlying disease, length of PICU stay, need for mechanical ventilation and death. Five criteria were used to classify the presence of bacterial infection at admission or during the PICU stay: new infiltrate on the chest radiograph, increased need for supplemental oxygen, fever (temperature of 38.5°C or greater), increased infectious parameters (C-reactive protein >40 mg/l and/or white blood cell count >15 × 10^9^/l) and positive bacterial culture (blood or sputum). For the purpose of this study, 'no bacterial infection', 'possible bacterial infection' and 'proven bacterial infection' were defined as the presence of fewer than two, two to three, and more than three of the above criteria, respectively.

For the analysis of continuous variables, an unpaired *t* test was used to compare means. Categorical variables were analyzed using a two-tailed Fisher's exact test.

## Results

Twenty-six PICU patients met the inclusion criteria. One patient was admitted with a positive RSV test result from an outside hospital, and no further testing was performed at our institution. Another patient diagnosed with a bacterial pneumonia was subjected to no virus tests, and insufficient material was obtained from a third patient to perform all respiratory tests. Consequently, 23 patients were included in the subsequent analysis.

The median age of the patients was 2.6 months, and the majority were referred from an outside hospital (Table 1). The PICU patients formed a heterogeneous group, with many patients having underlying conditions resulting from preterm birth or birth defects. Patients stayed in the PICU for a median of 10 days; 20 (87%) patients needed mechanical ventilation. Two patients in the study group died. One child suffered from bronchiolitis with progressive bronchospasms, which in the end could not be controlled. The second child had complex underlying conditions; after eliminating all treatable causes (including LRTI) the patient died from central hypoventilation.

Viral culture identified a respiratory virus in six PICU patients; RSV and adenovirus were detected in four and two patients, respectively (Table 2). The DIF assay appeared to be more sensitive to detect respiratory viruses than viral culture in our population, identifying a virus in 11 (48%) patients, including the six patients diagnosed by viral culture.

Real-time PCR detected a total of 33 respiratory viruses in 22 (96%) patients. All positive results found by conventional techniques were confirmed by real-time PCR. RSV was the single most common respiratory virus found and was detected in 16 (70%) patients. In addition, rhinovirus was found in six (26%) patients, and influenzavirus, adenovirus and coronavirus were each detected in three (13%) children. PIV-3 and hMPV were both found in one patient (4%). PIV-2 and PIV-4 and the atypical pathogens *Mycoplasma pneumoniae *and *Chlamydia pneumoniae *could not be detected in any of the LRTI patients.

In eight (35%) children more than one virus was detected. Six children had a dual infection, one had a triple, and one had a quadruple respiratory virus infection. RSV was detected in seven of the eight children infected with multiple viruses, including all three patients infected with a coronavirus. Of the 14 children with a single infection, nine had RSV. The other five patients had an infection with rhinovirus (*n *= 2), influenzavirus (*n *= 1), adenovirus (*n *= 1), or hMPV (*n *= 1).

No statistical differences were found between children with a single and multiple virus infection with respect to age, sex, gestational age, underlying disease, length of PICU stay, mechanical ventilation and the presence of bacterial infection.

Bacterial cultures were performed in 14 patients. In eight (57%) patients bacterial pathogens were identified (most commonly *Streptococcus pneumoniae*, *Moraxella catarrhalis*, *Haemophilus influenzae *and *Staphylococcus aureus*). The details for each patient regarding viral and bacterial infections, including length of stay, are presented in Additional data file 1. According to the predefined classification, six (26%) children in the study group had proven, 11 (48%) had possible and six (26%) had no bacterial infection. Of the six patients with a proven bacterial infection, two infections developed after more than 48 hours of mechanical ventilation. Overall, 19 (83%) patients received antibiotics during their stay on the PICU for a median of 7 days. Patients with a proven bacterial infection stayed in the PICU for a mean of 18 days versus 9 days for patients with possible or no bacterial infection (*P *= 0.026).

## Discussion

In the present study conventional methods (viral culture and DIF) were compared with real-time PCR for their ability to detect respiratory viruses in young children admitted to the PICU with LRTI. The use of real-time PCR increased the diagnostic yield from 48% to 96%, and all viruses found by conventional methods were confirmed by real-time PCR. Whereas with conventional methods no double infections were found, real-time PCR revealed multiple virus infections in 35% of patients.

The high diagnostic yield of PCR compared with conventional techniques is in agreement with the findings of others. When conducting a Medline search (1966 to September 2005) with the search terms LRTI, PCR, and RSV or respiratory viruses, we identified 11 hospital-based paediatric studies that compared PCR with conventional techniques for more than two respiratory viruses [8-11,16-22]. PCR was found to have excellent sensitivity in all studies, if conventional methods were considered the reference standard (0.91–1.00). In summary, these studies showed that PCR increased the diagnostic yield by by up to 25% as compared with conventional techniques. However, most studies selected patients on the basis of respiratory test results, rather than selecting patients by admission diagnosis. Exceptions include the studies conducted by Weinberg and coworkers [22] and Jennings and colleagues [9]. Weinberg and coworkers [22] tested samples from 668 hospitalized children and selected patients on admission diagnoses by the New Vaccine Surveillance Network. PCR assays were used to test for RSV, influenzaviruses and PIV 1–3. With viral culture 89 (13%) specimens were positive, as compared with 185 (28%) positive specimens with PCR. Jennings and colleagues [9] enrolled 75 hospitalized children using a case definition of acute respiratory infection, and performed viral diagnostics for a broad range of viruses. PCR identified 39 additional viruses, adding up to a total of 87 viruses found in 65 (87%) children [9].

The clinical relevance of detection of respiratory viruses such as rhinovirus and coronavirus is a matter of debate. Rhinovirus [23] and coronavirus [14] are well recognized as causes of the common cold. On the other hand, there is increasing evidence that they are also important causes of severe lower respiratory disease. Rhinovirus infections have been reported in elderly adults with LRTI or pneumonia [24,25] and in immunocompromised patients [26,27]. Coronavirus infection has also been reported in cases of LRTI [14,25,28,29]. Real-time PCR will provide insight into the role played by such viruses in the aetiology of LRTI.

The high yield of PCR compared with conventional techniques could theoretically be the result of contamination. However, with using real-time PCR the likelihood of false-positive results caused by carry-over contamination is reduced to a minimum. This is achieved by the use of modified nucleotides (dUTP) and uracil-DNA glycosylase (UNG) for control of contamination in the PCR-based amplification of (c)DNA as well as the closed-tube detection system [3]. In addition, during the procedures of DNA/RNA isolation and amplification, several negative controls are included to monitor for possible false-positive findings.

An additional advantage of real-time PCR over standard-format PCR is that it allows high-throughput screening of patient samples for the presence of many different pathogens. Our real-time PCR assays included testing not only for RSV, influenzaviruses and PIVs, but also for rhinoviruses, adenoviruses, the recently discovered hMPV [30], coronaviruses (OC43, 229E and the newly identified coronavirus NL63 [31]) and the atypical pathogens *Mycoplasma pneumoniae *and *Chlamydia pneumoniae*. Thus, real-time PCR allows for maximal detection of multiple viral and atypical infections in children with LRTI, with a negligible risk for false-positive results.

In the PICU patients studied, we found a high prevalence of multiple viral infections (35%), including triple and even quadruple infections. In previous studies the detection of multiple infections varied considerably, ranging from 0.6% to 27%. A low prevalence was found in studies using PCR for RSV, influenzaviruses and PIVs only [18,22]. In contrast, a high prevalence was found in studies that included a broad range of respiratory viruses (including rhinovirus, adenovirus, coronavirus and hMPV) [9]. Interestingly, in the latter study most coronaviruses were identified as part of multiple virus infections (three out of four), which is in accordance with our findings.

The prevalence of possible or proven bacterial infection in our study group was high (74%). The fact that almost half of the children were diagnosed with a possible bacterial infection indicates the difficulty of precluding this diagnosis. In addition, previous studies showed that viral infection may pave the way for bacterial infection [32]. The only study that compared viral diagnostic methods in a similar population of PICU patients did not report on the prevalence of bacterial infections, and so we can not compare this finding with those from other studies [16]. In contrast to the high prevalence of possible or proven bacterial infection found in our study group, we did not demonstrate infections with atypical pathogens such as *Mycoplasma pneumoniae *and *Chlamydia pneumoniae*. It can be speculated that atypical pathogens do not play an important role in children with severe LRTI.

The small study group included in our study represents a limitation; it did not allow us to find an association between clinical characteristics and multiple infections. Furthermore, the samples that were negative by conventional methods and positive by real-time PCR could not be confirmed using a true gold standard because such a standard for respiratory viruses does not exist. This is a problem encountered in all studies of respiratory viruses.

The finding that real-time PCR increases the yield of viral diagnoses for PICU patients with LRTI has implications for clinical practice. The rapidity and sensitivity of real-time PCR test results can help the clinician to initiate appropriate cohorting strategies to prevent other critically ill children from nosocomial viral infections. A reliable and rapid viral test result can also be taken into account when prescribing or discontinuing antibiotic treatment. Our study was not designed to determine when antibiotic treatment for LRTI in the PICU is warranted. The high percentage of possible or proven bacterial infections in our viral LRTI patients indicates that antibiotic treatment poses a dilemma in the PICU. The clinical impact of the high prevalence of multiple virus infections detected in our PICU remains unclear because they were not associated with different clinical characteristics. Further research with larger numbers of patients and age-matched control groups is needed to determine the real clinical impact of multiple infections and to determine whether the use of real-time PCR prevents unnecessary antibiotic treatment. However, real-time PCR offers a rapid, sensitive and highly reliable new technique that may improve our understanding of the epidemiology of severe LRTIs.

## Conclusion

Real-time PCR for a broad range of respiratory viruses was found to be highly sensitive in children with severe lower respiratory tract infection in the PICU. In addition, real-time PCR increased the diagnostic yield of positive samples by twofold compared with conventional methods (viral culture and DIF). Whereas conventional methods identified no multiple infections, real-time PCR found the prevalence of multiple infections to be 35%. Because real-time PCR is a rapid and sensitive technique, it is able to guide initial patient cohorting strategies and therapy in the PICU.

## Key messages

• 	Real-time PCR for the detection of respiratory viruses is a sensitive and reliable method in PICU patients with LRTI, and it increases the diagnostic yield compared with conventional methods.

• 	Real-time PCR allows detection of multiple viral infections (found in 35% of patients in this study) that are not identified using conventional methods.

• 	The rapidity and sensitivity of real-time PCR test results can help the clinician to initiate appropriate cohorting strategies to prevent other critically ill children from acquiring nosocomial viral infections.

## Abbreviations

DIF = direct immunofluorescence; hMPV = human metapneumovirus; LRTI = lower respiratory tract infection; PCR = polymerase chain reaction; PICU = paediatric intensive care unit; PIV = parainfluenzavirus; RSV = respiratory syncytial virus; RT = reverse transcriptase.

## Competing interests

The authors declare that they have no competing interests.

## Authors' contributions

TW directed the study design and writing of the report. JR collected samples, directed viral diagnostics and data analysis, and revised the report. NJ participated in the design of the study, directed the acquisition and interpretation of clinical data, and revised the report. AvL played a substantial role in conceiving and designing the study and revising the manuscript. AvdP performed viral diagnostics, collected and analyzed the data, and wrote the report. All authors read and approved the final manuscript.

## Supplementary Material

Additional File 1 A Word file containing a table that summarizes the viral and bacterial pathogens for each patient. Click here for file

## Figures and Tables

**Table 1:** Demographic and clinical characteristics of children with lower respiratory tract infection on admission to the PICU

Characteristic	Value
Demographics	
Age, median months (range)	2.6 (0.5–26.5)
Male	10 (43%)
Admissions from outside hospital	20 (87%)
Underlying conditions	
Preterm birth (<37 weeks)	11 (48%)
Underlying disease	11 (48%)
Pulmonary	3
Cardiac	4
Other	5
Severity	
ICU stay (days; median [range])	10 (2–33)
Mechanically ventilated at PICU	20 (87%)
Deaths due to LRTI	1

A total of 23 patients were included in the study. LRTI, lower respiratory tract infection; PICU, paediatric intensive care unit.

**Table 2:** Viruses identified by conventional methods and real-time PCR

Pathogen	Viral culture (*n *= 21)	Immunofluorescence (*n *= 22)	Real-time PCR (*n *= 23)
RSV A/B	4	7	16 (9)
Influenzavirus A/B	0	2	3 (1)
Rhinoviruses	0		6 (2)
Adenoviruses	2	2	3 (1)
Coronavirus OC43, 229E, NL63			3
hMPV			1 (1)
PIV 1/3	0	0	1
PIV 2/4	0	0	0
*Chlamydia pneumoniae*			0
*Mycoplasma pneumoniae*			0
Indeterminate		4	
Total positive	6^a^	11^a^	33

Numbers in parentheses indicate single infections. ^a^Single infections. hMPV, human metapneumovirus; PCR, polymerase chain reaction; PIV, parainfluenzavirus; RSV, respiratory syncytial virus.

## References

[B1] Shay DK, Holman RC, Newman RD, Liu LL, Stout JW, Anderson LJ (1999). Bronchiolitis-associated hospitalizations among US children, 1980–1996. JAMA.

[B2] Henrickson KJ, Hoover S, Kehl KS, Hua W (2004). National disease burden of respiratory viruses detected in children by polymerase chain reaction. Pediatr Infect Dis J.

[B3] Pang J, Modlin J, Yolken R (1992). Use of modified nucleotides and uracil-DNA glycosylase (UNG) for the control of contamination in the PCR-based amplification of RNA. Mol Cell Probes.

[B4] Henrickson KJ (2004). Advances in the laboratory diagnosis of viral respiratory disease. Pediatr Infect Dis J.

[B5] Adcock PM, Stout GG, Hauck MA, Marshall GS (1997). Effect of rapid viral diagnosis on the management of children hospitalized with lower respiratory tract infection. Pediatr Infect Dis J.

[B6] Barenfanger J, Drake C, Leon N, Mueller T, Troutt T (2000). Clinical and financial benefits of rapid detection of respiratory viruses: an outcomes study. J Clin Microbiol.

[B7] Woo PC, Chiu SS, Seto WH, Peiris M (1997). Cost-effectiveness of rapid diagnosis of viral respiratory tract infections in pediatric patients. J Clin Microbiol.

[B8] Gruteke P, Glas AS, Dierdorp M, Vreede WB, Pilon JW, Bruisten SM (2004). Practical implementation of a multiplex PCR for acute respiratory tract infections in children. J Clin Microbiol.

[B9] Jennings LC, Anderson TP, Werno AM, Beynon KA, Murdoch DR (2004). Viral etiology of acute respiratory tract infections in children presenting to hospital: role of polymerase chain reaction and demonstration of multiple infections. Pediatr Infect Dis J.

[B10] Kehl SC, Henrickson KJ, Hua W, Fan J (2001). Evaluation of the Hexaplex assay for detection of respiratory viruses in children. J Clin Microbiol.

[B11] Syrmis MW, Whiley DM, Thomas M, Mackay IM, Williamson J, Siebert DJ, Nissen MD, Sloots TP (2004). A sensitive, specific, and cost-effective multiplex reverse transcriptase-PCR assay for the detection of seven common respiratory viruses in respiratory samples. J Mol Diagn.

[B12] van Elden LJ, Nijhuis M, Schipper P, Schuurman R, van Loon AM (2001). Simultaneous detection of influenza viruses A and B using real-time quantitative PCR. J Clin Microbiol.

[B13] van Elden LJ, van Loon AM, van der Beek A, Hendriksen KA, Hoepelman AI, van Kraaij MG, Schipper P, Nijhuis M (2003). Applicability of a real-time quantitative PCR assay for diagnosis of respiratory syncytial virus infection in immunocompromised adults. J Clin Microbiol.

[B14] van Elden LJ, van Loon AM, van Alphen F, Hendriksen KA, Hoepelman AI, van Kraaij MG, Oosterheert JJ, Schipper P, Schuurman R, Nijhuis M (2004). Frequent detection of human coronaviruses in clinical specimens from patients with respiratory tract infection by use of a novel real-time reverse-transcriptase polymerase chain reaction. J Infect Dis.

[B15] van Doornum GJ, Guldemeester J, Osterhaus AD, Niesters HG (2003). Diagnosing herpesvirus infections by real-time amplification and rapid culture. J Clin Microbiol.

[B16] Akhtar N, Ni J, Stromberg D, Rosenthal GL, Bowles NE, Towbin JA (1999). Tracheal aspirate as a substrate for polymerase chain reaction detection of viral genome in childhood pneumonia and myocarditis. Circulation.

[B17] Bellau-Pujol S, Vabret A, Legrand L, Dina J, Gouarin S, Petitjean-Lecherbonnier J, Pozzetto B, Ginevra C, Freymuth F (2005). Development of three multiplex RT-PCR assays for the detection of 12 respiratory RNA viruses. J Virol Methods.

[B18] Erdman DD, Weinberg GA, Edwards KM, Walker FJ, Anderson BC, Winter J, Gonzalez M, Anderson LJ (2003). GeneScan reverse transcription-PCR assay for detection of six common respiratory viruses in young children hospitalized with acute respiratory illness. J Clin Microbiol.

[B19] Fan J, Henrickson KJ, Savatski LL (1998). Rapid simultaneous diagnosis of infections with respiratory syncytial viruses A and B, influenza viruses A and B, and human parainfluenza virus types 1, 2, and 3 by multiplex quantitative reverse transcription-polymerase chain reaction-enzyme hybridization assay (Hexaplex). Clin Infect Dis.

[B20] Freymuth F, Vabret A, Galateau-Salle F, Ferey J, Eugene G, Petitjean J, Gennetay E, Brouard J, Jokik M, Duhamel JF, Guillois B (1997). Detection of respiratory syncytial virus, parainfluenzavirus 3, adenovirus and rhinovirus sequences in respiratory tract of infants by polymerase chain reaction and hybridization. Clin Diagn Virol.

[B21] Gilbert LL, Dakhama A, Bone BM, Thomas EE, Hegele RG (1996). Diagnosis of viral respiratory tract infections in children by using a reverse transcription-PCR panel. J Clin Microbiol.

[B22] Weinberg GA, Erdman DD, Edwards KM, Hall CB, Walker FJ, Griffin MR, Schwartz B, New Vaccine Surveillance Network Study Group (2004). Superiority of reverse-transcription polymerase chain reaction to conventional viral culture in the diagnosis of acute respiratory tract infections in children. J Infect Dis.

[B23] Makela MJ, Puhakka T, Ruuskanen O, Leinonen M, Saikku P, Kimpimaki M, Blomqvist S, Hyypia T, Arstila P (1998). Viruses and bacteria in the etiology of the common cold. J Clin Microbiol.

[B24] Nicholson KG, Kent J, Hammersley V, Cancio E (1996). Risk factors for lower respiratory complications of rhinovirus infections in elderly people living in the community: prospective cohort study. BMJ.

[B25] Falsey AR, Walsh EE, Hayden FG (2002). Rhinovirus and coronavirus infection-associated hospitalizations among older adults. J Infect Dis.

[B26] Malcolm E, Arruda E, Hayden FG, Kaiser L (2001). Clinical features of patients with acute respiratory illness and rhinovirus in their bronchoal-veolar lavages. J Clin Virol.

[B27] Ison MG, Hayden FG, Kaiser L, Corey L, Boeckh M (2003). Rhinovirus infections in hematopoietic stem cell transplant recipients with pneumonia. Clin Infect Dis.

[B28] Folz RJ, Elkordy MA (1999). Coronavirus pneumonia following autologousbone marrow transplantation for breast cancer. Chest.

[B29] Pene F, Merlat A, Vabret A, Rozenberg F, Buzyn A, Dreyfus F, Cariou A, Freymuth F, Lebon P (2003). Coronavirus 229E-related pneumonia in immunocompromised patients. Clin Infect Dis.

[B30] van den Hoogen BG, de Jong JC, Groen J, Kuiken T, de Groot R, Fouchier RA, Osterhaus AD (2001). A newly discovered human pneumovirus isolated from young children with respiratory tract disease. Nat Med.

[B31] van der Hoek L, Pyrc K, Jebbink MF, Vermeulen-Oost W, Berkhout RJ, Wolthers KC, Wertheim-van Dillen PM, Kaandorp J, Spaargaren J, Berkhout B (2004). Identification of a new human coronavirus. Nat Med.

[B32] Bakaletz LO (1995). Viral potentiation of bacterial superinfection of the respiratory tract. Trends Microbiol.

