# Supplementary material for: Diagnostic value of real-time polymerase chain reaction to detect viruses in young children admitted to the paediatric intensive care unit with lower respiratory tract infection
Source: Crit Care. 2006 Apr 12;10(2):R61. doi: 10.1186/cc4895 (PMC1550925; doi:10.1186/cc4895)
Supplement: Additional File 1 — A Word file containing a table that summarizes the viral and bacterial pathogens for each patient. [file cc4895-S1.doc]

**Online table A.** Viral and bacterial pathogens and bacterial infection specified per patient.

| Patient | Age (months) | Underlying Disease | ICU stay (days) | Bacterial Infection | Bacterial  Culture | Viral Culture | Immuno-  fluorescence | Real-time PCR |
| --- | --- | --- | --- | --- | --- | --- | --- | --- |
| 1 | 1.7 | Preterm birth | 14 | possible | *S. pneumoniae, M. catarrhalis, H. influenzae*^c^ |  |  | RSV |
| 2 | 1.6 | Preterm birth, long-term oxygen | 33 | proven | *S. pneumoniae, S. aureus*^c^ |  | RSV | RSV |
| 3 | 26.5 | Preterm birth, BPD | 15 | no |  |  |  | RSV |
| 4^a^ | 0.8 | Spina bifida, Arnold Chiari type II malformation | 6 | no |  |  | indeterminate | RSV, Rhinovirus |
| 5 | 0.8 |  | 18 | possible | *M. catarrhalis*^c^ |  | indeterminate | RSV |
| 6 | 2.6 |  | 4 | possible |  |  | RSV | RSV |
| 7 | 2.3 |  | 8 | no |  |  |  |  |
| 8 | 2.7 | Preterm birth, IVH | 16 | proven | *H. influenza*^d^ | RSV | RSV | RSV |
| 9 | 2.4 | Multiple congenital disorders, microcephaly | 3 | no |  |  |  | Parainfluenzavirus 3, Rhinovirus |
| 10 | 1.2 | Preterm birth | 13 | possible |  | RSV | RSV | RSV |
| 11 | 8 | Asthma | 24 | proven | *K. oxytoca, H. influenza*^c^ | Adenovirus | Adenovirus | RSV , Adenovirus, Coronavirus, Rhinovirus |
| 12 | 3.2 | Preterm birth, s/p ASD, VSD, PDA | 15 | proven | *S. aureus*^c^ |  | Influenzavirus | RSV, Influenzavirus |
| 13 | 2.6 |  | 10 | possible |  |  | RSV | RSV, Influenzavirus, Adenovirus |
| 14 | 11.4 | Preterm birth | 4 | possible |  | Adenovirus | Adenovirus | Adenovirus |
| 15^b^ | 2.3 |  | 7 | possible | *S. pneumoniae, M. catarrhalis*^c^ |  | Influenzavirus | Influenzavirus |
| 16 | 8.8 | ASD, VSD | 10 | possible |  | RSV | RSV | RSV |
| 17 | 0.5 |  | 2 | no |  | RSV | RSV | RSV, rhinovirus |
| 18 | 2.6 |  | 11 | possible |  |  | indeterminate | RSV, coronavirus |
| 19 | 4.4 | Preterm birth, s/p NEC, colostoma | 9 | possible |  |  | indeterminate | Rhinovirus |
| 20 | 4.9 | Preterm birth | 12 | proven | *S. pneumoniae, M. catarrhalis, H. influenza*^c^ |  |  | human Metapneumovirus |
| 21 | 2.1 | Preterm birth | 10 | possible |  |  |  | RSV, Coronavirus |
| 22 | 2.1 | Preterm birth, ASD, PDA, TI, PHT | 10 | no |  |  |  | RSV |
| 23 | 5.8 |  | 10 | proven |  |  |  | Rhinovirus |

^a^ died, cause: LRTI

^b^ died, cause: underlying disease

^c^ sputum culture

^d^ blood culture

Abbreviations: ICU: intensive care unit, RSV: respiratory syncytial virus, BPD: bronchopulmonary dysplasia, IVH; intraventricular haemorrhage, s/p: status post, ASD atrial septal defect, VSD: ventricular septal defect, PDA: patent ductus arteriosus, NEC: necrotizing enterocolitis, TI: tricuspid insufficiency, PHT: pulmonary hypertension.
